# Advanced resuscitative interventions in refractory out-of-hospital cardiac arrest: a retrospective cohort study of a physician-led pre-hospital team

**DOI:** 10.1016/j.resplu.2026.101331

**Published:** 2026-04-17

**Authors:** N. Kruit, B. Burns, I. Ferguson, K. Fowler, D. Tian, M. Dennis, the PRECARE trial group, Neil Ballard, Jimmy Bliss, Jackie Buckthought, Katie Ellis, Christopher Ennis, Ian Ferguson, Karel Habig, Geoff Healey, Malcolm Lau, Helen Oliver, Christopher Partyka, Alexander Peters, Cliff Reid, Christopher Wilkinson

**Affiliations:** 1 GSA-HEMS, NSW Ambulance, Bankstown Aerodrome, Sydney, New South Wales, Australia; a NSW Ambulance, Faculty of Medicine and Health, University of Sydney, Department of Perioperative Medicine, Westmead Hospital, Australia; b GSA-HEMS, NSW Ambulance, Bankstown Aerodrome, Sydney, New South Wales, Australia; c Liverpool Hospital and Retrieval Physician, Aeromedical Operations, New South Wales Ambulance, South West Clinical School, University of New South Wales, Sydney, New South Wales, Australia; d NSW Ambulance, Australia; e Department of Anaesthesia and Perioperative Medicine, Westmead Hospital, Sydney, Australia; f The George Institute for Global Health, University of New South Wales, Sydney, Australia; g Department of Cardiology, Royal Prince Alfred Hospital, Faculty of Medicine and Health, University of Sydney, Australia

## Introduction

High performance CPR with a focus on uninterrupted chest compressions is the foundation of cardiac arrest resuscitation and has been shown to improve outcomes.^1^, ^2^ However, this uniform approach to resuscitation may not take into consideration individual patient factors, characteristics or response to treatment.

Anatomical variability in the position of the heart and great vessels may influence the efficacy of chest compressions, particularly when the left ventricular outflow tract, aortic root or ascending aorta lie beneath the recommended compression position.^3^, ^4^, ^5^, ^6^ potentially rendering the application of compressions ineffective with limited to no forward flow and poor tissue perfusion. These observations highlight the potential importance of an individualised approach to resuscitation guided by real-time physiological feedback to ensure effective forward flow with compressions.^7^, ^8^ Coronary perfusion pressure and diastolic blood pressure (DBP) are commonly measured in resuscitation research but not commonly used in routine out of hospital cardiac arrest (OHCA) care in a targeted manner during resuscitation. There is emerging literature that these parameters can be used to augment resuscitation efforts in a physiological endpoint targeted fashion, whilst enabling assessment of intervention effectiveness. This strategy may be associated with improved resuscitation outcomes.^9^, ^10^

In order to provide personalised, targeted CPR, advanced diagnostic tools traditionally only provided in the hospital setting, are required. Often by the time of arrival to hospital, the window for using these tools and performing meaningful intervention has already passed. There is little evidence demonstrating the successful application of targeted CPR in the pre-hospital setting. Pre-hospital critical care doctors have additional skills, interventions, diagnostic tools and medications that are able to be delivered on-scene during cardiac arrest. The use of these interventions may augment resuscitation management and improve outcomes.

The objective of this paper is to provide descriptive analysis of a dedicated medical cardiac arrest team (MCAT) case mix and the frequency of advanced diagnostic and therapeutic modalities not routinely used in the pre-hospital setting to aid in physiologically guided resuscitation.

## Methods

### Study design

A retrospective observational cohort study of consecutive non-traumatic OHCA patients attended by the MCAT. Approval was granted by the Sydney Local Health District Human Research Ethics Committee (HREC Project Number: X23-0150 & 2023/ETH00767), with a waiver of patient consent.

### Setting

New South Wales Ambulance (NSWA) is the single emergency medical service (EMS) across New South Wales (population 8.2 million across 800,000 km^2^) and continues resuscitation on approximately 3325 patients per year. Cardiac arrests are assigned the highest priority and responded to by a minimum of two paramedic teams. All paramedics are trained in basic and advanced life support and follow established Australian and international guidelines for this. Clinical skill levels are tiered, the highest tier of paramedic, an intensive care paramedic (ICP) is also invariably tasked to a cardiac arrest. ICPs provide the additional skills of endotracheal intubation (during cardiac arrest) and intraosseous access as well as additional pharmacological therapies. There is a statewide cardiac reperfusion system, with centres that provide protocolised primary percutaneous coronary intervention for ST-elevation myocardial infarction (STEMI) 24/7, post cardiac arrest intensive care and ECPR may be available in office hours in selected centres. A dedicated MCAT, consisting of two specialist prehospital and retrieval medicine doctors and one critical care paramedic, commenced regular operations three days a week on January 1st 2024 as part of the PRECARE trial, examining the feasibility of pre-hospital extracorporeal cardiopulmonary resuscitation (ECPR) delivery by an existing pre-hospital medical team.^11^ The team responds from a location prospectively determined by geospatial analysis as the optimal location to access a majority of the Sydney metropolitan area within 45 min travel time^12^ between the hours of 0700 and 1900.

### Participants

The team was dispatched to all non-traumatic cardiac arrest patients aged <70 yrs where the arrest was believed to be witnessed and resuscitation had been initiated.

Exclusion criteria:

• Age <18 years
• Cases where MCAT arrived on scene and did not intervene due to futility or advanced age
• Eligible patient’s receiving pre-hospital ECPR- this patient group included: witnessed arrest with bystander CPR commenced within 5 min, age <70 years and initial shockable rhythm.

#### Study size

All consecutive eligible patients attended during the study period were included. No formal sample size calculation was performed due to the descriptive nature of the study.

### MCAT composition and interventions

#### MCAT Composition

MCAT comprises of ten senior physicians and five critical care paramedics employed by Aeromedical Operations, NSW Ambulance. The doctors are specialists in emergency medicine or anaesthesia with a sub-speciality in pre-hospital and retrieval medicine. Team members are trained in point of care ultrasound transthoracic echocardiography (TTE). One clinician is an accredited TOE practitioner. In addition to providing ECPR, the team is equipped to provide advanced cardiac arrest resuscitation utilising invasive haemodynamic monitoring, additional pharmacology including thrombolysis and vasopressors and transoesophageal (TOE) guided CPR.

#### Scene processes

Advanced life support by the initial paramedic response continues as per international guidelines. On arrival of the MCAT, eligible patients receive ECPR as published elsewhere.^11^ Patients suitable for continued resuscitation, but ineligible for ECPR receive advanced diagnostics and haemodynamic interventions that may include invasive blood pressure monitoring via a femoral arterial line, a targeted diastolic blood pressure of >30 mmHg (measured off the first systolic inflexion point of the arterial line waveform), resuscitative TOE (if accredited practitioner was present), transthoracic echocardiography (TTE), thrombolysis, point of care arterial blood gas measurement, and optimisation of ventilation strategy. These interventions are used to optimise chest compression position, maximise coronary artery perfusion, and to identify and treat reversible pathologies. There was no pre-specified protocol for achievement of the targeted DBP. Decisions as to dose and method of adrenaline (infusion or bolus) and/or vasopressin was left to the treating clinician.

### Primary outcome

Frequency of advanced resuscitation interventions delivered at MCAT treated out of hospital cardiac arrests

### Secondary outcomes

1. The frequency and timing of advanced interventions performed by the MCAT including:
  (a) Advanced airway and ventilation
  (b) Intra-arrest invasive arterial pressure monitoring
  (c) Diastolic blood pressure target >30 mmHg
  (d) Transoesophageal echocardiography and targeted compression placement
  (e) Point of care blood gas analysis
2. Proportion of patients achieving sustained ROSC (>20 min) on arrival at hospital
3. Differences in patient demographics, OHCA case characteristics, MCAT interventions and key resuscitation time intervals between sustained ROSC and non-ROSC groups.

### Data collection and analysis

All cardiac arrests attended by the MCAT had resuscitation monitoring data captured in real-time. Standard monitoring is undertaken using the Zoll X-series monitor (Zoll Medical Corporation, Tokyo, Japan) and included end tidal CO_2_ waveform capnography (mmHg), plethysmography (SpO_2_), electrocardiogram (ECG), invasive arterial blood pressure (mmHg). Monitoring is applied as per usual practice, and a monitor print out of this record is routinely attached to the patient’s medical record at the end of the patient encounter. Key timings including time of collapse, dispatch of team, time of arrival of team and departure of team are routinely recorded on the case sheet.

A copy of the patient’s case sheet, monitor print out and an electronic database entry are all stored on the NSWA Aeromedical database (AirMaestro, Avinet). All resuscitative TOE study loops are timestamped, recorded and stored on the ultrasound machine (Venue Go, GE Healthcare) hard drive with the TOE study loops exported and later uploaded to the patient’s medical record.

Relevant pre-hospital data was collected and entered in an electronic case report form using REDCap (Vanderbilt University), a web-based secure electronic database hosted by Sydney Local Health District. Data from patients attended and treated by the MCAT over the study period were reviewed and abstracted including demographic, dispatch times, cardiac arrest characteristics, pre-hospital interventions and disposition.

#### Bias

Interventions were applied at clinician discretion based on scene factors, introducing potential treatment selection bias. Consecutive case inclusion was used to reduce selection bias.

### Statistical analysis

Descriptive statistics are presented as median with interquartile range for continuous variables and frequencies with percentages for categorical variables, unless otherwise described. Between group differences were assessed using either Student’s *t*-test or the Mann-Whitney test as appropriate for continuous variables, and the chi-squared test or Fisher’s exact test as appropriate for categorical variables. Normality was assessed with Lilliefors test. The level of statistical significance was 0.05. No multivariable modelling was performed due to the exploratory and descriptive nature of the study. Analysis was performed using R statistical software version 3.5.2 (R Core Development Team, Vienna, Austria).

## Results

### Participants

**Fig. 1:**
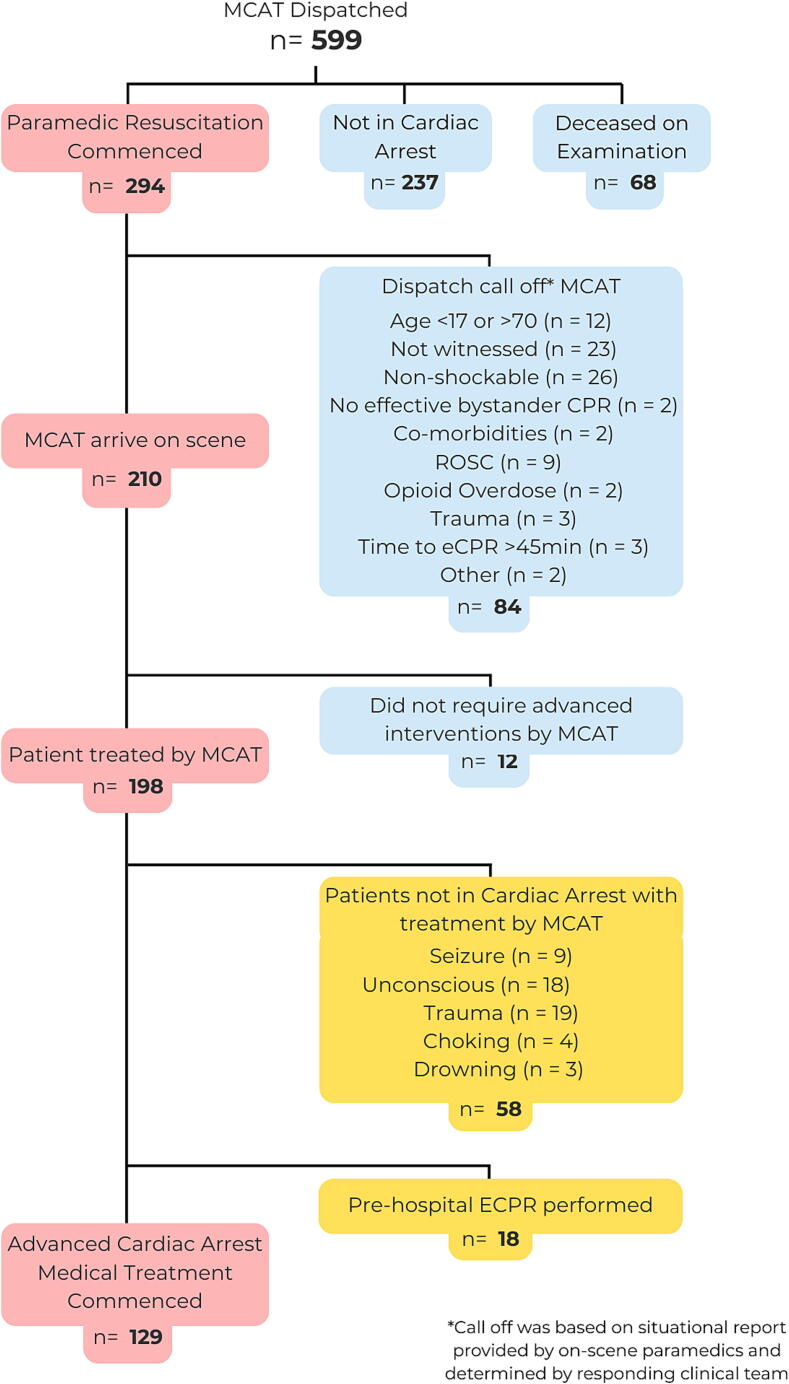
Consort diagram demonstrating cases MCAT attended.

MCAT was dispatched to 599 cases and arrived on scene to 210 cases over an 18-month period, spanning 180 days of operation. Advanced diagnostics and haemodynamic interventions including invasive arterial line monitoring, TOE and point of care testing, were provided to 147 patients who remained in cardiac arrest. Eighteen patients receiving pre-hospital ECPR were excluded from this analysis (Fig. 1), leaving an MCAT cohort of 129 patients.

### Baseline characteristics

The median age was 54 years (range 44–62), 96 (74%) were male, 61 (47%) patients had an initial shockable rhythm on attendance of the first paramedics. Ninety (70%) arrests were witnessed and 76 (59%) had CPR commenced within five minutes of collapse (Table 1, Fig. 2). Sustained ROSC was achieved in 49% of all included patients.

**Table 1:** Demographic data and treatment prior to arrival of MCAT.

	**All** **(*n* = 129)**	**ROSC on arrival to hospital** **(*n* = 63)**	**No ROSC** **(*n* = 66)**	**Missing**
**Baseline demographics**				
Male: *n* (%)	96 (74)	48 (76)	48 (73)	0
Median age (yrs):	54 (44–62)	54 (44–61)	55 (44–62)	0
Bystander witnessed: *n* (%)	90 (70)	46 (73)	44 (67)	0
Paramedic witnessed: *n* (%)	11 (9)	6 (10)	5 (8)	0
Bystander CPR commenced within 5 min of collapse: *n* (%)	76 (59)	41 (65)	35 (53)	0
Median time to first paramedics on scene (mins):	10 (6–12)	9 (6–11)	10 (6–13)	1 (1)
**Initial rhythm on EMS arrival**				
Total non-shockable: *n* (%)*				
• Asystole	40 (31)	12 (19)	28 (42)	0
• PEA	26 (20)	12 (19)	14 (21)	0
• ROSC	2 (2)	2 (3)	0	0
**Total shockable: *n* (%)**#				
• VF	57 (44)	35 (56)	22 (33)	0
• VT	4 (3)	2 (3)	2 (3)	0
Median no. automated external defibrillations (AED) prior to first ambulance arrival: (range)	0 (0–0)	0 (0–0)	0 (0–0)	0
Median first recorded ETCO_2_ (mmHg): (range)	36 (23–59)	33 (23–48)	38 (22–61)	42 (33)
Median no. paramedic delivered defibrillations prior to MCAT arrival: (range)	1 (1–3)	2 (1–3)	1 (0–3)	49 (38)
Median time to first paramedic defibrillation (min): (range)		4 (1–38)	5 (1–25)	

* Percentage derived from total non-shockable.

# Percentage derived from total shockable.

**Fig. 2:**
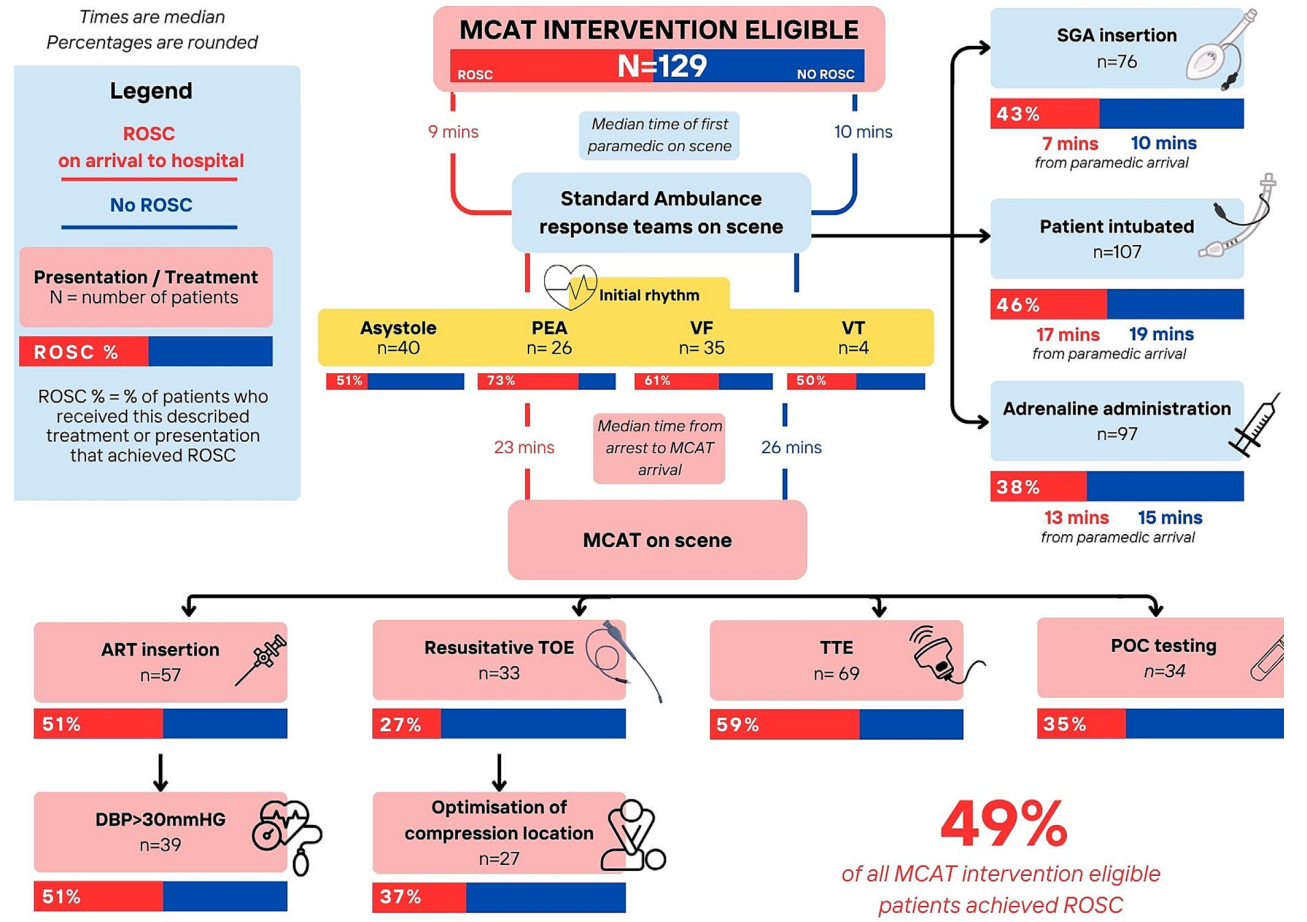
MCAT Interventions.

### Process and timing characteristics

The median time from arrest to arrival of MCAT was 25 min (range 20–34). The median time from MCAT arrival to sustained ROSC was 14 min (0–24) with 20 patients achieving sustained ROSC before MCAT interventions had been commenced.

On exploratory post-hoc analysis this was shorter in the sustained ROSC group at 23 min (range 17–31) versus 26 min (range 21–38 mins) in the no ROSC group (*p* = 0.016).

### Interventions delivered

Resuscitative TOE was performed in 33(26%) patients. Compression position was modified in 27 (82%) patients, 11 (37%) of whom achieved sustained ROSC on arrival to hospital. Three patients had a large embolus identified in the main pulmonary artery. The median time from collapse to TOE was 35 mins (range 6–51 min). Transthoracic echocardiography (TTE) was performed in 69 (54%) patients to identify reversible causes and differentiate low flow PEA from cardiac standstill (Fig. 2). Pericardial tamponade resulting in needle pericardiocentesis was performed in one patient.

#### Invasive arterial line monitoring and point of care testing

Targeted haemodynamic resuscitation with invasive femoral arterial line monitoring was performed in 57 (44%) patients. A diastolic pressure target >30 mmHg was achieved in 39 (68%) patients, 20 (51%) had sustained ROSC (Fig. 2). Point of care testing was performed in 34/118 patients (29%) 12 (35%) had sustained ROSC.

#### MCAT critical decision making

Of the patients who remained in refractory cardiac arrest and would ordinarily have been conveyed to hospital by paramedics, the MCAT determined resuscitation to be futile and pronounced life extinct on scene in 19 (26%) thereby avoiding hospital transport.

### Comparative analysis

Between-group comparisons of sustained ROSC versus non-ROSC patients are presented in table. No multivariable modelling was performed due to the descriptive and exploratory nature of the study.

## Discussion

We describe our experience of a physician-led pre-hospital cardiac arrest team in providing advanced physiologically guided interventions to selected refractory OHCA patients. Additional interventions not available to existing paramedics were provided in 129 cases, with 49% achieving sustained ROSC on arrival to hospital. Invasive haemodynamic monitoring enabled the achievement of DBP target >30 mmHg in the majority of monitored patients and TOE frequently resulted in the modification of compression position. Whilst this study was not designed to demonstrate efficacy, it does demonstrate the operational feasibility of delivering invasive, targeted resuscitation strategies in the pre-hospital environment. These interventions enable real time adjustment of resuscitation and may assist in augmentation of myocardial blood flow, improving ROSC and resuscitation success^10^, ^13^, ^14^, ^15^, ^16^, ^17^, ^18^, ^19^ with current resuscitation guidelines recommending haemodynamic targeted CPR where feasible.^20^, ^13^

Consistent with our previous work,^21^ TOE-guided compression identified ineffective compressions in a large proportion of patients, and position adjustments were associated with ROSC in approximately 1/3 of patients where it was applied. Prior studies have demonstrated an association between TOE-identified compression position over the left ventricle and increased likelihood of ROSC.^6^, ^22^, ^23^, ^24^ This modality permits continuous cardiac imaging during ongoing chest compressions without interrupting CPR and can be used to assess compression depth and location. Delaying application of TOE guided optimisation until hospital arrival may forfeit a critical time-sensitive opportunity to assess and potentially optimise haemodynamic parameters during resuscitation.

When invasive blood pressure monitoring was established, sustained ROSC occurred in 51% of patients maintaining a DBP exceeding 30 mmHg. Whether DBP represents a modifiable therapeutic target or a prognostic marker of resuscitation remains uncertain.^25^ However, the application of these advanced strategies demonstrates the feasibility of deploying advanced haemodynamic monitoring and clinician-led haemodynamic titration in the prehospital environment. While end-tidal CO_2_ is considered a non-invasive marker of cardiac output and CPR quality,^7^, ^13^ it does not directly reflect coronary perfusion. The relationship between diastolic blood pressure, myocardial blood flow and improved resuscitation success is well documented^14^, ^15^, ^16^ with a diastolic pressure of >25 mmHg associated with an improved chance of ROSC.^10^, ^13^, ^17^, ^18^, ^25^ Haemodynamic-directed CPR strategies, including real time assessment of chest compression quality and vasopressor titration, have been shown to improve intra-arrest physiology and potentially survival outcomes^26^, ^27^ and is recommended in current clinical guidelines,^13^ yet remains rarely described in the pre-hospital setting.^25^ Our findings suggest that invasive blood pressure monitoring is operationally achievable outside hospital and may provide a more complete physiological picture to non-invasive methods in determining CPR efficacy.

Despite its potential, physiologically based, goal directed resuscitation in the pre-hospital setting remains limited,^21^ likely reflecting the paucity of prehospital critical care teams who possess the tools and capability to perform this, balanced against the logistical challenges of providing effective, timely, non-interrupted resuscitation. We have demonstrated that it is feasible to perform advanced interventions in a time pressured austere environment. Of note, the no-ROSC group that included a higher incidence of poor prognostic indicators were more likely to be prolonged refractory arrests and therefore received more advanced interventions implemented by the MCAT, raising the possibility of reverse survivorship bias i.e. more interventions were provided as the arrests were more refractory. Moreover, it is likely that any therapeutic benefit of advanced therapies will be greater earlier during a cardiac arrest resuscitation. The heterogenous application of interventions, prevented a time-based analysis of intervention versus ROSC in this study, but should be considered in future studies.

The overall utility of pre-hospital medical teams on outcomes for OHCA has been recently assessed by an ILCOR-led systematic review of over 1 million patients.^28^ The study reported a statistically significant association with improved survival on hospital arrival, discharge, and 30-day neurological outcomes,^28^ although the evidence remains limited by the absence of randomised data and a low level of certainty. A further systematic review conducted by ILCOR reported that increasing EMS clinician exposure to OHCA was associated with improved 30-day survival.^29^, ^30^ Our study was not designed to assess the efficacy of the MCAT over standard ambulance response and the lack of comparator data in our study prevents any assessment of such. Whilst our ROSC on hospital arrival rate is promising for a prolonged refractory cardiac arrest cohort with a high proportion of non-shockable rhythms, unwitnessed arrests, and delays in bystander CPR.^25^, ^31^, ^32^, ^33^, ^34^ further investigation with more rigorous study designs, survival outcome follow-up and earlier intervention to delineate causation are required and planned.

Given this, and the association between earlier MCAT attendance and obtaining ROSC, it is possible that the addition of a dedicated cardiac team with increased on-scene resources, advanced resuscitation interventions, and higher case volume by the experienced dedicated team contributed to this finding. In addition, bringing hospital-based resuscitation techniques to the patient, supplementing the high-quality advanced life support provided by paramedics, mitigates the deterioration in resuscitation quality that has been shown to occur with intra-arrest transport.^35^ Further investigation with more rigorous study designs, survival outcome follow-up and earlier intervention to delineate causation are currently being planned by our group.

The provision of what was once thought of as traditionally hospital-based resuscitation techniques in the pre-hospital environment by a medical team can potentially improve equity of access to advanced resuscitation and reduce the need for intra-arrest transport, which may compromise resuscitation. We have previously demonstrated no significant benefit of intra-arrest transportation,^36^ where challenges in accessing these advanced therapies likely limited the ability to demonstrate a treatment effect. Moreover, current resuscitation guidelines recommend OHCA patients be cared for in dedicated cardiac arrest centres to improve survival outcomes,^20^, ^37^ though randomised trials examining cardiac arrest centres have thus far reported no benefit.^38^ In developing these centres, caution must be exercised not to further exacerbate inequities of care.^37^ A roving medical team can mitigate this inequity with the ability to support longer transport distances ensuring the patient is delivered to the facility that will provide the most appropriate level of care.^39^

## Limitations

This was a retrospective, descriptive study without a comparator group, precluding any causal inference regarding the effectiveness of the MCAT model or individual interventions. We were unable to provide survival to hospital discharge or 30-day follow up data as this is not routinely collected as part of our OHCA registry process. However, this retrospective study was to describe the interventions and is not designed, nor powered for clinical endpoints. Future studies are being designed to address this. The application of interventions was inconsistent due to no set clinical protocol. However, the study was to describe the interventions rather than a strict protocolised approach. Interventions were more frequently used in prolonged or refractory arrests, introducing resuscitation time and reverse survivorship bias. Data completeness was variable due to the operational constraints of pre-hospital resuscitation. Only one clinician could perform pre-hospital TOE, limiting generalizability.

## Conclusion

We describe the preliminary experience of a dedicated pre-hospital medical cardiac arrest team providing advanced diagnostic and therapeutic procedures. Prospective, and ideally comparator studies, are required to assess both team and intervention effects on survival.

## Data sharing

The data supporting the findings of this study are available from the corresponding author upon reasonable request.

## Declaration of generative AI and AI-assisted technologies in writing process.

AI was not utilised in the writing process.

## CRediT authorship contribution statement

**N. Kruit:** Writing – original draft, Methodology, Investigation, Formal analysis, Data curation, Conceptualization. **B. Burns:** Writing – review & editing. **I. Ferguson:** Writing – review & editing. **K. Fowler:** Writing – review & editing, Visualization. **D. Tian:** Writing – review & editing, Formal analysis. **M. Dennis:** Writing – review & editing, Methodology.

## Funding

MD is supported by a National Heart Foundation future leaders fellowship grant and a New South Wales Office of Health and Medical Research, early career grant.

MD is supported by a Post-Doctoral Scholarship (Ref: 105849) from the 10.13039/501100001030National Heart Foundation of Australia. 10.13039/501100001516The National Heart Foundation had no role in the study design, collection, analysis or interpretation of the data nor in writing of the data and submission of the article.

This study received no funding.

## Declaration of competing interest

The authors declare no conflicts of interest.
